# Targeted plant improvement through genome editing: from laboratory to field

**DOI:** 10.1007/s00299-020-02655-4

**Published:** 2021-01-21

**Authors:** Dragana Miladinovic, Dulce Antunes, Kubilay Yildirim, Allah Bakhsh, Sandra Cvejić, Ankica Kondić-Špika, Ana Marjanovic Jeromela, Hilde-Gunn Opsahl-Sorteberg, Antonios Zambounis, Zoe Hilioti

**Affiliations:** 1 Institute of Field and Vegetable Crops, Novi Sad, Serbia; 2 MED, FCT, Universidade do Algarve, Faro, Portugal; 3 Department of Molecular Biology and Genetics, Faculty of Sciences, Ondokuzmayıs University, Samsun, Turkey; 4 Department of Agricultural Genetic Engineering, Faculty of Agricultural Sciences and Technologies, Nigde Omer Halisdemir University, Nigde, Turkey; 5 Faculty of Biosciences, Norwegian University of Life Sciences, Ås, Norway; 6 Department of Deciduous Fruit Trees, Institute of Plant Breeding and Genetic Resources, ELGO-DEMETER, Naoussa, Greece; 7 Institute of Applied Biosciences, CERTH, Thessaloniki, Greece

**Keywords:** Genome editing, Breeding, Plants, Improvement, Traits, Disease resistance

## Abstract

**Key message:**

**This review illustrates how far we have come since the emergence of GE technologies and how they could be applied to obtain superior and sustainable crop production.**

**Abstract:**

The main challenges of today’s agriculture are maintaining and raising productivity, reducing its negative impact on the environment, and adapting to climate change. Efficient plant breeding can generate elite varieties that will rapidly replace obsolete ones and address ongoing challenges in an efficient and sustainable manner. Site-specific genome editing in plants is a rapidly evolving field with tangible results. The technology is equipped with a powerful toolbox of molecular scissors to cut DNA at a pre-determined site with different efficiencies for designing an approach that best suits the objectives of each plant breeding strategy. Genome editing (GE) not only revolutionizes plant biology, but provides the means to solve challenges related to plant architecture, food security, nutrient content, adaptation to the environment, resistance to diseases and production of plant-based materials. This review illustrates how far we have come since the emergence of these technologies and how these technologies could be applied to obtain superior, safe and sustainable crop production. Synergies of genome editing with other technological platforms that are gaining significance in plants lead to an exciting new, post-genomic era for plant research and production. In previous months, we have seen what global changes might arise from one new virus, reminding us of what drastic effects such events could have on food production. This demonstrates how important science, technology, and tools are to meet the current time and the future. Plant GE can make a real difference to future sustainable food production to the benefit of both mankind and our environment.

## Introduction

Conventional breeding has enabled breeders to produce improved varieties of many crops and has led to increased food security and crops with higher yield and tolerance to biotic and abiotic stress, as well as increased nutrient content. However, in the era of the changing climate and greater consumer demands, breeders are still facing increasing challenges expected to be overcome. Projected changes in climate are expected to have far-reaching impacts on agricultural production, affecting future food production. The rising temperatures, droughts or floods in a certain geographical area, as well as new pests and diseases, will stress plants and demand new varieties and changed production systems differently in different geographic regions. Current global food systems are based on a few cereal crops and elite varieties feeding both animals and humans. Although seemingly efficient, it is not resilient to sudden changes in yield shocks posed by environmental changes or changed trading due to changed demands or changed financial market balance. By 2050, the FAO estimates that the food demand will have increased over 60% and it will have needed 50% more energy and 40% more water to feed the 10 billion people on the planet Earth. Furthermore, climate changes could result in a global temperature rise with detrimental effects which might be associated with disease outbreaks threatening the crop production and compromising the quality of the harvested products (Raza et al. 2019).

**Fig. 1:**
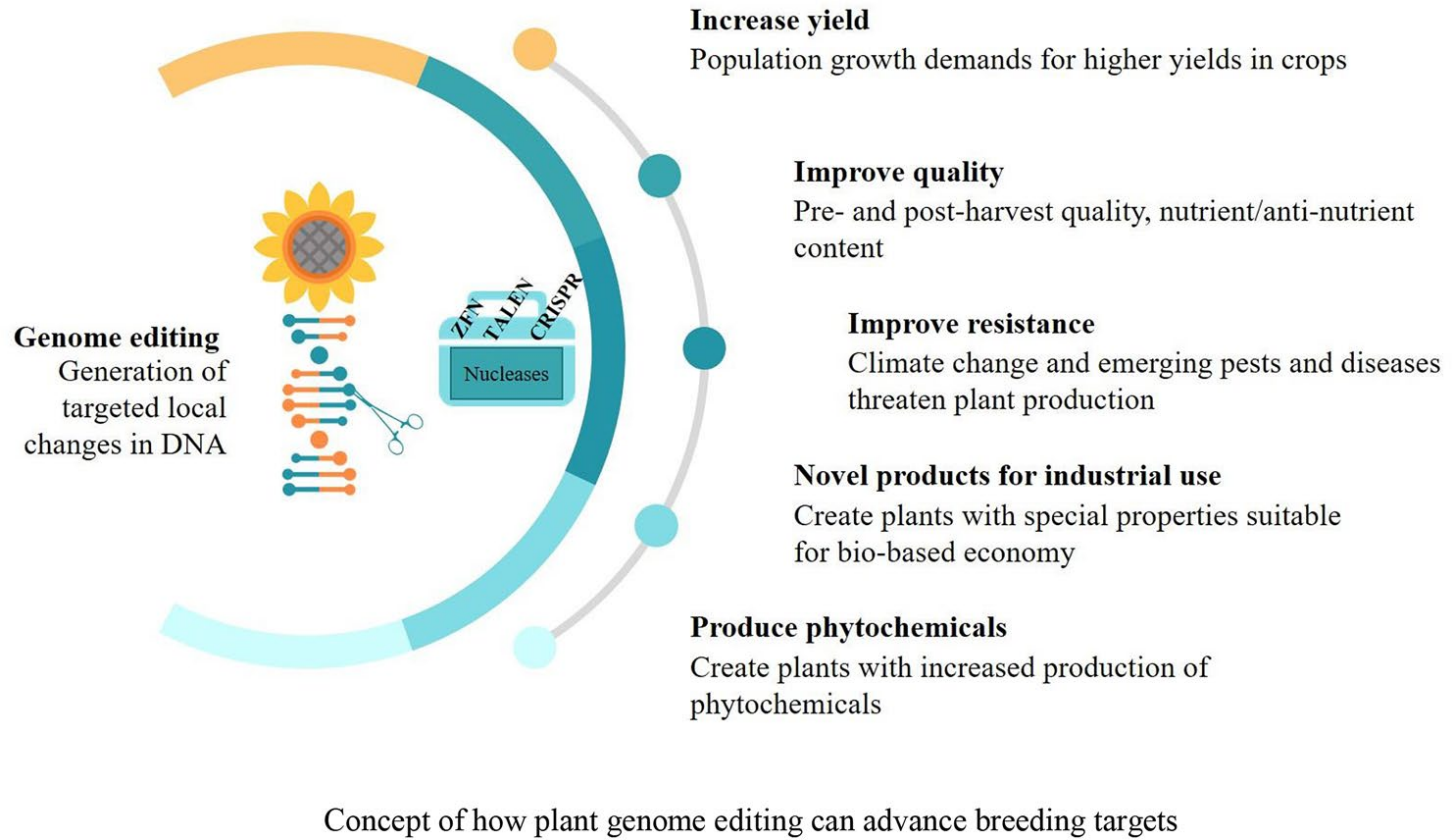
Concept of how plant genome editing can advance breeding targets

Genome editing provides new tools for the rational design of crops with improved traits (Fig. 1). These tools could enable faster production of new crop varieties, better adapted to any changes, whether environments or different consumer preferences around the world, and their transfer “from laboratory to field”. Transgene-free genome editing (GE) technologies have opened a new era in plant precision breeding by providing better tools to increase (agro) biodiversity resources by means of trait engineering. In this review, we stress different aspects and potential applications of genome editing for plant improvement through precision breeding.

## Genome editing toolbox

Genome editing involves the ability to introduce specific changes in DNA, such as the insertion, deletion or replacement of DNA through sequence-targeted recombination that has increased the speed, ease, and reproducibility of making local DNA changes. Although GE technologies have progressed rapidly and have successfully been applied to a wide range of cells and organisms, there is a considerable variation in their efficiency of cleavage at the target site. This variation could be explained by different modes of action of these technologies, as well as their differences in target site specificity, modular assembly and construction methods. In spite of variation in their efficiency, these technologies open up new opportunities for efficient plant improvement and some of the resulting changes, whether made through conventional breeding, gene transfer, genome editing or natural spontaneous mutation, cannot be distinguished.

The in vitro replication of specific DNA sequences by polymerase chain reaction (PCR) and the studies on the mechanisms of DNA double-strand breaks (DSBs) repair have allowed plant scientists to develop tools for targeted mutagenesis in plants. One of the first tools in the GE toolbox was the zinc finger nucleases (ZFNs). ZFNs are chimeric proteins with a DNA cleavage domain of FokI restriction endonuclease (a bacterial protein) and an array of three or four zinc fingers that were originally identified in sequence-specific eukaryotic transcription factors. The first eukaryotic sequence-specific transcription factor to be characterized was found to have zinc-binding repeats in its DNA-binding domain (Miller et al. 1985). Each zinc finger module recognizes three to four bases of sequence. These targeted hybrid restriction enzymes were developed by Srinivasan Chandrasegaran and his team (Kim et al. 1996) and require dimerization mediated by the FokI cleavage domain for their function (Smith et al. 2000). A ZFN pair recognizes two adjacent DNA sequences on opposite strands with combined 18-nucleotide recognition specificity. In 2005, Dana Carroll and his team successfully achieved targeted mutagenesis in *Arabidopsis* by using ZFNs (Lloyd et al. 2005).

The second tool to edit genes, the transcription activator-like effector nucleases (TALENs), is composed of Transcription Activator-Like Effectors (TALEs) and the FokI endonuclease. TALE proteins are expressed by plant bacterial pathogens to manipulate host gene transcription and promote successful infection. Like ZFNs, TALENs function as pairs in a similar way to create a break at a specific DNA sequence recognized by the TALE domain. TALENs for DNA recognition use a tandem array of 16 (or more) nearly identical protein modules, each of which targets one nucleotide at the DNA target site, making thus TALENs highly specific (Boch et al. 2009; Christian et al. 2010).

Meganucleases or homing endonucleases are sequence-specific endonucleases recognizing for cleavage long sequences (typically 18–30 base pairs) that occur only once in any given genome and for this reason are rare-cutting enzymes. They generate DSBs, and the site-specific I-SceI is the prototypical meganuclease that has been used as a tool for genome engineering. For genome editing purposes, thousands of meganucleases have been redesigned and mutants created with new specificities (reviewed in Daboussi et al. 2015).

CRISPR/Cas (clustered regularly interspaced short palindromic repeats/CRISPR-associated protein) is an easier and more efficient genome editing tool than the engineered ZFNs and TALENs. It is based on the adaptive immune system in bacteria and archaea, enabling organisms to respond and eliminate invading viruses and plasmids (Jinek et al. 2012; Cong et al. 2013). This system consists of three components: the Cas9 nuclease from *Streptococcus pyogenes* (or similar alternatives), crRNA, and tracrRNA. The combination of the crRNA and tracrRNA into a single synthetic single guide RNA (sgRNA) has resulted in a simplified two-component reagent that is now widely used to introduce targeted double-stranded breaks in genomic DNA. The sgRNA contains approximately 20-base-long sequence complementary to the target DNA, which guides Cas9 to the right genomic location.

All above-described methods offer, with varying degrees of success, easy-to-design and cost-effective tools for precise and efficient plant genome editing. In recent years, they have also emerged as powerful tools that could be used for targeted plant improvement through directed mutagenesis and creation of varieties with increased stress resilience and enhanced quality convenient for different uses (Table 1).

**Table 1:** Genome editing in plants for targeted improvement of different traits

Trait	Plant/crop	GE tool used	Description	Reference
Yield	Maize	CRISPR / Cas9	Drought tolerance	Shi et al. ( 2017 )
	Rice	CRISPR / Cas9	Identification of high-yield genes	Li et al. ( 2016a ), Huang et al. ( 2018 )
		CRISPR / Cas9	Early maturation	Li et al. ( 2017a )
		CRISPR / Cas9	Grain weight	Xu et al. (2016)
	Tomato	ZFN	Phenotypic variability	Hilioti al. (2016)
Disease resistance	Banana	CRISPR / Cas9	Resistance against banana streak virus	Tripathi et al. (2019)
	Cacao	CRISPR / Cas9	Resistance against *Phytophthora tropicalis*	Fister et al. (2018)
	Tomato	CRISPR / Cas9	Resistance against powdery mildew	Nerkasov et al. (2017)
		CRISPR / Cas9	Broad spectrum disease resistance	Thomazella et al. (2016)
	Wheat	CRISPR / Cas9	Resistance against powdery mildew	Zhang et al. (2017b)
Quality-food	Peanuts	LANGUAGES	Increased oleic acid content	Wen et al. ( 2018 )
	Potato	LANGUAGES	Reduced levels of acrylamide	Clasen et al. ( 2016 )
	Rapeseed	CRISPR / Cas9	Increased oleic acid content	Okuzaki et al. ( 2018 )
	Rice	CRISPR / Cas9	High amylose content	Sun et al. ( 2017 ), Zhang et al. ( 2018b )
		ZFN	Low starch content	Jung et al. ( 2018 )
	Soybean	LANGUAGES	Altered fatty acids levels	Haun et al. ( 2014 ), Demorest et al. ( 2016 )
	Tomato	CRISPR / Cas9	Increased lycopene content	Li et al. ( 2018a )
		CRISPR / Cas9	Fruit ripening	Ito et al. ( 2015 )
		CRISPR / Cas9	Increase of y-aminobutyric acid	Li et al. ( 2018c )
		CRISPR / Cas9	Parthenocarpic plants	Nonaka et al. ( 2017 )
		CRISPR / Cas9	Long shelf life	Ueta et al. ( 2017 )
		ZFN	Increased antioxidant content, low oxalic acid (anti-nutrient), high fructose to glucose ratio	Yu et al. ( 2017 ), Gago et al. ( 2017 )
	Wheat	CRISPR / Cas9	Low gluten content	Sanchez-Leon et al. (2018)
Quality-feed	Alfalfa	LANGUAGES	Reduced lignin content	USDA (2017)
	Maize	Meganuclease	Increased level of starch in leaves and	USDA (2015)
	Sorghum	CRISPR / Cas9	Increased protein digestibility and quality	Li et al. (2018b)
Non-food	Camelina	CRISPR / Cas9	Altered fatty acids composition	Aznar-Moreno and Durrett (2017), Ozseyhan et al. (2018)
	Cotton	CRISPR / Cas9	Lignocellulosic fibre formation and elongation	Zhu et al. (2018), Li et al. ( 2017b )
	Miscanthus	CRISPR / Cas9	Lignin reduction and content manipulation	Golfier et al. (2019)
	Poplar	CRISPR / Cas9	Lignin reduction and content manipulation	Zhou et al, ( 2015 ), Wan et al. 2017 ; Wang et al. (2017), Xu et al. ( 2017 ), Yang et al. (2017 )
	Rice	CRISPR / Cas9	Change in lignin composition	Takeda et al. (2018)
	Sugarcane	LANGUAGES LANGUAGES	Lignin reduction Increased saccharification efficiency	Jung and Altpeter ( 2016 ), Kannan et al. 2018)
	Switchgrass	CRISPR / Cas9	Lignin reduction	Park et al. ( 2017)

## Yield improvement through genome editing

Crop yield and consequently food production are highly influenced by complex interactions among climatic and soil conditions, abiotic and biotic stresses, and crop management practices. These interactions may have either negative or positive impact on plant growth and productivity (Jovičić et al. 2019; Kondić-Špika et al. 2019; Marjanović Jeromela et al. 2019). Therefore, the combined effect of different abiotic stresses (Song et al. 2014; Paul et al. 2019), as well as biotic and abiotic stresses (Pautasso et al. 2012; Pandey et al. 2015) on plant physiology and development, has extensively been investigated. Because biotic stresses have a huge role in potential yield loss and contribute to 15% of global declines in food production (Oerke 2005), studies with disease forecasting models for different regional climatic scenarios have been performed (Oldenburg et al. 2009; Caffarraa et al. 2012; Jevtić et al. 2017). With all this knowledge in mind, different strategies for crop production increase have been developed so far. A significant improvement of yield and other important traits was achieved by using conventional breeding tools for more than 50 years (Hristov et al. 2009; Mladenov et al. 2011). However, tackling climate change will largely depend on the new breeding techniques which possess the ability to develop desired traits more precisely and quickly than conventional breeding methods. CRISPR/Cas9-based genome editing is one of these techniques, and it has been adopted in many crop species so far (Ricroch et al. 2017). In many studies, it was demonstrated that CRISPR/Cas9 is an efficient technology for improving crop yield by knocking out the genes that negatively regulate yield-related traits (Zhang et al. 2018a; Lu et al. 2018; Liu et al. 2017b, a; Li et al. 2016a). A multiplexing GE strategy has been employed for trait pyramiding, and the following results have been obtained: enhanced grain size and weight in rice (Xu et al. 2016), early heading in rice (Li et al. 2017a), as well as increased kernel weight in wheat (Zhang et al. 2016). In a recent study, Huang et al. (2018) identified 57 genes controlling yield-related traits in 30 varieties of the Green Revolution phenotype known as “miracle rice” by combining genome sequencing and CRISPR/Cas9 technique and created knockout mutants of those 57 genes. Phenotyping of these mutants enabled identification of several genes that are crucial for regulating yield-related traits in rice. This new approach can be very useful in examining complex quantitative traits, including yield.

Genome editing has also been utilized to increase crop stress tolerance and to modify some important development and metabolic processes (Pandey et al. 2015; Rodriguez-Leal et al. 2017; Razzaq 2019a, b; Xu et al. 2019). These alterations in horticultural plants and crops were used as indirect ways to improve their performance and yield in different environments.

## GE for improved disease resistance

Plants are plagued by numerous phytopathogens, including fungi, bacteria, and viruses which cause severe crop yield losses worldwide (Dangl et al. 2013; Fisher et al. 2012). Upon pathogen challenge in plants a signal transduction system is induced orchestrating the establishment of defence responses. The plant innate immunity has a fine-tuned two-tiered immune perception system that initially involves the activation of pattern recognition receptors (PRRs), which perceive pathogen-associated molecular patterns (PAMPs) to initiate a basal defence response called pathogen-triggered immunity (PTI) (Thomma et al. 2011). In addition, intracellular nucleotide-binding site (NBS) and leucine-rich repeat (LRR)-containing cytoplasmic receptors (NLRs) initiate the effector-triggered immunity (ETI). The ETI system activates complex plant immune reactions for defence against pathogens (Win et al. 2012; Zhang et al. 2017a).

Plant defence mechanisms are constantly evolving to early respond against new diseases (Whitham et al. 2016; Zambounis et al. 2016). However, adaptation of pathogens to altering environment conditions happens quite faster than in plants. Innovative GE techniques including CRISPR-associated protein 9 (CRISPR/Cas9) system, TALENs and ZFNs, the first developed GE tool, as well as LAGLIDADG homing endonucleases have already been employed for engineering disease resistance in crops (Borrelli et al. 2018; Langner et al. 2018; Dong et al. 2019; Mushtaq et al. 2019). These GE techniques can enable precise and efficient development of plant varieties resistant to a broad-spectrum of pathogens, by modification of the genes that confer susceptibility to a given pathogen.

In general, the CRISPR/Cas9 system has high efficiency and simplicity, although there is the risk of off-target effects, allowing the development of plant species with enhanced disease resistance (Mushtaq et al. 2019). Identifying pathogen-resistance genes and gene target sites for CRISPR/Cas9-based editing will enable functional testing of large numbers of variants. This approach has most frequently been used on the mildew resistance locus O (*MLO*), using RNA-guided Cas9 endonuclease (Nekrasov et al. 2017). CRISPR/Cas9 has also been used in rice for the induction of mutagenesis in the promoter of host-susceptibility (*S*) OsSWEET family of putative sugar transporter genes, *OsSWEET14* and *OsSWEET11*, conferring tolerance against bacterial blight (Jiang et al. 2013). Recently, Thomazella et al. (2016) have reported that employment of CRISPR/Cas9 system enhanced disease resistance in tomato plants against different pathogens by inducing mutation in downy mildew resistance 6 (*SlDMR6-1*) gene. In grape, a *Botrytis cinerea*-responsive WRKY52 transcription factor has been targeted by the CRISPR/Cas9 system and the transgenic plants showed biallelic mutations less sensitive than the monoallelic mutants (Wang et al. 2018). In tree crops, the employment of transient leaf transformation targeting the non-expressor of pathogenesis-related 3 (*NPR3*) gene, which is a suppressor of the immune system in *Theobroma cacao*, has led to improved resistance to *Phytophthora tropicalis* (Fister et al. 2018).

The application of CRISPR/Cas9 technology for disease resistance is among the most applicable GE approaches in agricultural research (Borrelli et al. 2018). The current scientific knowledge of the molecular mechanisms underlying numerous plant–microbe interactions has contributed in choosing candidate genes to be edited through GE approaches. Targeting a single gene whose inactivation might lead to disease resistance can be technically less challenging (Borrelli et al. 2018). Broad-spectrum disease resistance has been achieved by targeting mainly specific *S* genes in many crops (Das and Rao 2015). These genes have emerged as the best candidates for engineering disease resistance, as they are often conserved among plant species and have the potential to be more durable in the field (Huibers et al. 2013).

The current knowledge of molecular mechanisms regulating plant–pathogen interactions would undoubtedly facilitate the employment of GE technologies in crop plants by the repression and activation of genes related to disease resistance. In order to increase the efficiency of GE approaches and avoid unexpected off-target mutations, rigorous design of the editing tool has to be performed (Borrelli et al. 2018). Main concerns for targeting and deploying GE approaches for broad-spectrum and durable resistance are the following: (i) There must be fundamental scientific knowledge about which gene(s) to modify and which type of modification to perform in these genes. For example, discovery of novel plant immune receptors and major virulence factors would enrich the repertoire and the pool of candidate deployable genes for GE. Comparative genomics approaches such as resistance gene enrichment sequencing (RenSeq) can also be employed to rapidly identify genomic variants in defence-related genes that are linked to disease phenotypes (Jupe et al. 2014). (ii) Field tests are necessary for the evaluation of agronomic fitness, the durability of the disease resistances and the agronomic management of the edited crops (Borrelli et al. 2018). Particularly, durability could be achieved by targeting several resistance genes, multiple metabolic and immune pathways induced downstream of NLRs whose resistance would be more difficult to break down rapidly. This multiplexing approach becomes more challenging with increasing plant host ploidy levels, as, for example, in hexaploid wheat (A, B, and D genomes), where three *MLO* gene alleles would need to be modified at once (Borrelli et al. 2018).

## Genome editing for improved food quality

The nutrient content of plants can have significant impact on nutritional status and human health. Food quality of plant-derived products is a combined outcome of the macronutrients, micronutrients, and phytochemicals, freedom from anti-nutrients and non-essential minerals or phytochemicals; and organoleptic attributes such as taste, flavour, aroma, appearance, texture, storage, and stability. Therefore, the concept of food quality embraces differentiated products for different end-uses. GE approaches allow targeted modifications, which, for example, in oilseed crops may be to create novel oil types by modifying their fatty acid profile or to improve the nutritional profiles or storage of fruit and vegetables. One of the first CRISPR’d crops that is expected to hit the market is waxy corn, a variety with edited deletion of the endogenous waxy gene *Wx1*, which encodes the endosperm’s granule-bound starch synthase responsible for making amylose, that resulted in elevated content of amylopectin and reduced levels of amylose (Waltz 2018). Corn starch with increased amylopectin content can have positive effects on the quality of frozen and canned food, by improving freeze–thaw properties in frozen food and making canned food creamier.

In plants, common nutritional targets include the modification of fatty acid composition and the enhancement of the antioxidant nutritional quality such as carotenoids, particularly lycopene, and flavonoids as well as the reduction of anti-nutrients. In tomato, a detailed characterization of eight ZFN-based mutant lines of LEAFY COTYLEDON1-LIKE4 (*L1L4*) transcription factor generated through a transient expression of ZFNs in seeds revealed increased soluble solids content, which is of prime importance in tomato fruit and a breeding target affecting flavour and nutritional value. Mutant lines enriched in *β*-carotene and antioxidants, ascorbic acid or succinic acid, were produced. Notably, the reduced content of the anti-nutrient oxalic acid in several mutant fruits suggests that *L1L4* gene regulates the accumulation of this compound in tomato during fruit development (Gago et al. 2017). A CRISPR/Cas9 system in tomato targeted genes in carotenoid metabolic pathway and achieved a 5.1-fold increase in the lycopene content in tomato fruit (Li et al. 2018a). In addition, tomatoes with increased *γ*-aminobutyric acid (GABA) levels have been produced by targeting two glutamate decarboxylase (*GAD*) genes, *GAD2* and *GAD3*, encoding a key enzyme in GABA biosynthesis. In this case, the CRISPR/Cas9 system created plants that produced tomatoes with 1.5- to tenfold higher GABA content, an amino acid that enhances the blood pressure-lowering function of tomato fruit (Nonaka et al. 2017). Generation of potato (*Solanum tuberosum*) varieties with undetectable levels of reducing sugars and reduced levels of acrylamide (a carcinogen) in processed chips was achieved though transient expression of TALENs designed to target the vacuolar acid invertase gene (*Vlnv)* (Clasen et al. 2016). In another work on potato, the granule-bound starch synthase gene, *GBSS*, that catalyses one of the enzymatic steps of starch synthesis, was mutated via CRISPR/Cas9. The mutated lines showed decreased levels of amylose and increased the amylopectin/amylose ratio (Andersson et al. 2017). In sorghum (*Sorghum bicolor*) grains, storage proteins called kafirins form protein bodies with poor digestibility. Kafirins are mostly composed of α-kafirins and encoded by the *k1C* family of highly similar genes. Li et al. (2018b) produced mutants with reduced kafirin levels and improved quality and digestibility of proteins using GE approach to target the *k1C* genes.

Rice (*Oryza sativa*) is the staple food for over half of the world's population and nutritional improvements of this species may have great impact on the human population. Utilization of ZFNs in this crop induced mutations in starch synthase IVa gene (*SSIVa)* that encodes a soluble starch synthase involved in starch biosynthesis pathway. Generation of transgenic plants revealed low starch contents and dwarf phenotypes (Jung et al. 2018). Furthermore, CRISPR/Cas9 system was used to introduce a loss-of-function mutation into the *Waxy* gene in two widely cultivated elite japonica varieties, resulting in a reduced amylose content and converted the rice into glutinous ones (Sun et al. 2017; Zhang et al. 2018b). Other important cereal, wheat, contains gluten proteins that are not tolerated by individuals with celiac disease. A CRISPR/Cas9 system targeted a conserved region adjacent to the coding sequence for the 33-mer in the *α*-gliadin genes and produced plants with low gluten levels in seed kernels (Sánchez-León et al. 2018). Similarly, it was reported that the zein proteins have been reduced by 12.5% in kernels by disrupting a maize *MADS* gene (GRMZM2G059102) that activates zein gene promoters (Qi et al. 2016).

Soybean (*Glycine max*) oil is used in applications ranging from cooking and frying to industrial products. Change of individual fatty acids content in soybean oil could contribute to its increased shelf-life and frying stability, as well as improvement of its nutritional value. Soybean varieties with high oleic acid were created using TALEN technology to target the genes coding fatty acid desaturase 2 enzyme, *FAD2-1A* and *FAD2-1B*. The oil profile of the mutant seeds meets the soybean industry’s demand as the oleic acid increased fourfold (from 20 to 80%), while linoleic acid decreased from 50 to less than 4% (Haun et al. 2014). Working on the same breeding target, another TALEN-mediated approach was used to introduce combined mutations within two fatty acid desaturase genes *FAD2-1A*, *FAD2-1B*, and one fatty acid desaturase 3 gene, *FAD3A,* in order to stack quality traits in soybean. The resulting mutant lines had oleic acid levels above 80% and linoleic and linolenic acid levels below 3% compared to wild-type oil (Demorest et al. 2016). Okuzaki et al. (2018) used CRISPR/Cas9 system to modify *FAD2* gene, which encodes an enzyme that catalyses the desaturation of oleic acid, in *Brassica napus* cv. Westar. The mutated lines had increased content of oleic acid in seeds, compared to the wild-type. In peanut (*Arachis hypogaea* L.), mutant lines with a 0.5–twofold increase in the oleic acid content were produced by targeted mutagenesis in the conserved coding sequence of *FAD2* gene by TALENs indicating that TALEN-mediated targeted mutagenesis can be used to increase the oleic acid content in edible peanut oil (Wen et al. 2018).

Plant cultivars with improved post-harvest quality are of significant importance for the reduction of food loss which is now estimated as 1/3 of the total production. Food loss reduction will contribute to improved sustainability of food production as it will also reduce the use of production inputs and land use. In tomato, the CRISPR/Cas9 system was used to target the indoleacetic acid-induced protein 9 (*IAA9*) gene, which controls parthenocarpy, the production of seedless fruit without prior fertilization (Ueta et al. 2017). In the same species, ZFN-mediated mutagenesis of seeds by transient electroporation-based transformation with over 65% efficiency targeted the *LIL4* gene, a master transcription factor encoding the *β*-subunit of the trimeric complex NF-Y. *LIL4* mutant lines had interesting agronomic traits such as earliness in flowering, variation in fruit size, colour, and shape (Hilioti et al. 2016). CRISPR/Cas9-mediated mutagenesis of the tomato (*Solanum lycopersicum*) RIPENING-INHIBITOR (*RIN*) gene, which encodes a MADS-box transcription factor regulating fruit ripening led to RIN-protein defective mutants with incomplete-ripening of fruits and reduced red colour pigmentation compared to wild-type fruit (Ito et al. 2015). In tomato, the CRISPR/Cas9 system delivered by the *Agrobacterium tumefaciens*-mediated *ALC* (alcobaca) gene mutagenesis in the presence of the homologous repair template. The resulting mutants produced tomatoes with prolonged shelf life (Yu et al. 2017). The long shelf life is a critical trait for the quality of fresh fruit, and it is one of the main objectives in breeding programs as it influences fruit storage and shelf life. Tomato has mostly been studied as a classic climacteric model species with fleshy fruits and the molecular basis of fruit ripening and softening has been studied extensively. The *RIN* and COLORLESS NONRIPENING (*CNR*) genes encode transcription factors involved in fruit ripening, and affect ripening in many fruit species, either climacteric or non-climacteric (Matas et al. 2009). Nevertheless, it has been shown that although it can improve shelf life, incomplete fruit ripening has adverse effects on organoleptic characteristics and nutritional quality. Thus, the challenge for ripening control is to modify the levels of gene expression sufficiently to extend shelf life without compromising the quality and sensory attributes (Matas et al. 2009).

## Alteration of lignocellulosic biomass by genome editing for improved feed quality

Highly condensed coverage of lignified tissue on cell wall polysaccharides in forage crops physically separates digestive hydrolytic enzymes of ruminants from carbohydrate source of lignocellulosic biomass. Consequently, lignification limits digestibility of animals, decreases energy yields, and increases overall cost of animal feeding. Therefore, several bioengineering and GE studies have been carried out on forage plants (maize, sorghum, rice, and alfalfa) to reduce lignin with a concurrent increase in cellulose content (Nair and Lee 2015; Barros et al. 2018; Zhenga et al. 2018). CRISPR/Cas9-based mutagenesis has successfully been applied to several forage crops for stable mutations in genes related to lignin biosynthesis. Takeda et al. (2018) compared CRISPR and RNAi techniques on rice mutants that harbour frameshift mutations in the p-coumaroyl ester 3′-hydroxylase (*C3′H*) gene, which is involved in both chlorogenic acid and lignin biosynthesis. In contrast to the RNAi-derived *C3′H*-knockdown mutants, the CRISPR-derived knockouts were severely dwarfed and sterile. The results of the study clearly indicated the impacts of *C3′H* suppression on lignin composition and on the assembly of other cell wall components in rice. Such structural alterations in rice were reported to be highly useful for enhancements in biomass digestibility and saccharification. The same research group investigated the suppression effect of coniferaldehyde 5‐hydroxylase (*OsCAld5H1*) gene, which modulates syringyl (S)/guaiacyl (G) lignin composition ratio, on lignin structure of rice. Loss-of-function mutants clearly demonstrated alteration of S/G subunit in lignin composition of rice cell wall (Takeda et al. 2019). Miyamoto et al. (2019) used CRISPR technology to generate lignin-enriched transgenic rice via targeted mutagenesis of the transcriptional repressor OsMYB108. In another study, *CAD2* double mutants, deficient in cinnamyl alcohol dehydrogenase (*CAD*), which encodes a key enzyme in lignin biosynthesis, were successfully created in rice with the same GE technique. Cell wall analysis of the *CAD2* mutants demonstrated altered lignification in rice and synergistic increase in saccharification efficiency via loss of function mutation (Matsumoto 2018). Pectin methyltransferase-deficient mutant rice was also generated by using CRSPR-mediated loss of function on acyltransferase 3 (*OsAT3*) and acyltransferase 4 (*OsAT4*). The result of both mutants indicated the effect of gene suppressions on pectin methyltransferase activity by a significant reduction in conversion of monolignols into corresponding lignin conjugates. CRISPR efficiency was also tested and successfully optimized for other forage crops such as maize (Svitashev et al. 2016; Armarego-Marriott 2020), grass (Liu et al. 2018) alfalfa (Gao et al. 2018; Curtin 2018 ), sorghum (Liu et al. 2019), wheat (Kumar et al. 2019), and soybean (Liu et al. 2019). In switchgrass, Park et al. (2017) targeted a key enzyme (4CL) in monolignol biosynthesis with CRISPR technology and produced plants with thinner cell walls. Remarkably, these plants had reduced lignin content by 30% and increased glucose and xylose release by 11% and 32% compared to wild type, respectively.

## Targeted plant improvement for non-food uses

Cellulose and hemicellulose are the main sources of sugar in the cell wall and the most valuable part of lignocellulosic biomass for the production of fuels, industrial chemicals and materials (Qian 2014). However, utilization of this sugar source in lignocellulosic materials has several restrictions due to protective coverage of lignin on them, which permits limited surface area for enzymatic and chemical hydrolysis (Ge et al. 2018). The manipulation of lignin composition and reduction of its content in plant cell wall improved suitability of lignocellulosic biomass for pulp, paper and textile industries as well as biofuel and easily digestible forage production (Häggman et al. 2013; Verma and Dwivedi 2014; Capstaff and Miller 2018). Various genetic and molecular techniques have been applied on lignocellulosic biomass to reduce lignin content and change its composition by down-regulating/knocking-out the lignin biosynthetic genes and regulatory transcription factors.

Until recently, alteration of lignocellulosic biomass for several plant species has mostly been carried out by bioengineering technologies such as T-DNA gene insertion-mutation, expression of antisense RNA, sense expression, co-suppression, and interference RNA (RNAi) (Zhao and Dixon 2014; Wang et al. 2015; Yang et al. 2019). However, such types of gene silencing methods have the risk of concomitant silencing of closely related gene family members that cause misinterpretations of the results and camouflaging the actual effects of individual gene silencing (Morgens et al. 2016). Widely used RNAi technology usually does not fully eliminate gene products (protein/enzyme), but only knockdown its expression. This unstable down-regulation is another important limitation of these gene silencing technique (Boettcher and McManus 2015; Zhou et al. 2015; Takeda et al. 2018). Another important shortcoming of these technologies is their dependency to stable gene transfer on the plant genome and creation of transgenic plants (Voelker et al. 2010; Van Acker et al. 2014; Zhou et al. 2015; Chutyser et al. 2018). All these problems can now easily be overcome by the use of new genome engineering methods for targeted genetic manipulation, such as ZNF, TALEN, and CRISPR (Häggman et al. 2013; Verma and Dwivedi 2014). Especially CRISPR system has become the most popular gene editing tool for several plant species, due to its low cost, simplicity and rapidness. CRISPR technology has made stable knockouts possible for specific target genes without insertion of foreign genetic material transfer into plant genome (Liu et al. 2017a). Therefore, it is possible to alter original base pair arrangement within plant genome without making it transgenic (Clifton-Brown et al. 2019). These bio-editing methods have started to be utilized for editing lignin biosynthetic genes to create mutant plants with reduced lignin content and better lignocellulosic properties (Gao 2018; Chanoca et al. 2019).

Carbohydrate polymers found in lignocellulosic biomass are used in pulp and paper industry. Due to the association of cellulose microfibrils with the condensed coverage of lignin, chemical delignification of wood is essential to remove lignin and produce high-quality paper with better brightness and whiteness (Chutyser et al. 2018). Chemical delignification requires expensive chemicals harmful for polysaccharide components of wood and for the environment due to toxic pollutants (Wang et al. 2018). To avoid these problems, GE could effectively be used to reduce lignin content and alter its composition in woody plants to improve quality of pulping, increase wood extractability, and reduce mill effluents (Verma and Dwivedi 2014). CRISPR-based gene knockout and silencing approach was effectively utilized to strongly down-regulate genes functional in lignin biosynthetic pathway in poplar species. The first stably CRISPR-based genome-edited poplar with high efficiency was reported by Fan et al. (2015). Bioinformatics tools to simplify GE were then developed quickly for heterozygous poplar species (Xue and Tsai 2015; Xue et al, 2015). Lignin biosynthesis via phenylpropanoid metabolism and cell wall traits are the main targets for the GE studies in poplar. CRISPR-Cas9 mutational efficiency was tested on lignin and flavonoid biosynthesis in the woody perennial *P. tremula* × *alba* by disrupting three 4-coumarate:CoA ligase genes (*4CL1*, *4CL2* and *4CL5*) (Zhou et al. 2015). Based on the results, *4CL1* and *4CL2* play a primary role in lignin and flavonoid biosynthesis. Mutations in *4CL1* gene revealed a reduction in lignification, whereas *4CL2* gene proved to be involved in chlorogenic acid production in leaves. In the same poplar study, CRISPR/Cas9-mediated mutagenesis in *4CL* gene revealed a 20% reduction in lignin content and a 30% decrease in S/G ratio. Importantly, each independent *4CL1* line developed uniform reddish-brown wood, a phenotype associated with lignin deficiency. In previous RNAi-based studies, *4CL1* suppression created patchy wood discoloration due to the unstable nature of RNAi-mediated gene silencing method (Voelker et al. 2010; Van Acker et al. 2014). CRISPR-based knockout studies targeting the MYB transcription factors in poplar were also studied to reduce lignin content. These studies revealed a negative regulatory role of some MYBs on the phenylpropanoid metabolism and secondary cell wall biosynthesis (PtoMYB156, PtoMYB115 and PtoMYB170), while some others increased proanthocyanidin biosynthesis (PtoMYB156and PtoMYB57), lignification (PtoMYB156 and PtoMYB170), and flavonoid accumulation (PtrMYB57 and PtoMYB115) (Wan et al. 2017; Wang et al. 2017; Xu et al. 2017; Yang et al. 2017). In poplar, brassinosteroid biosynthetic *PtoDWF4* gene knockout plants generated by CRISPR significantly decreased biomass production indicating the important role of the gene in secondary cell wall synthesis and wood formation (Shen et al. 2018). CRISPR-based knockouts for BRANCHED 1 (*BRC1-1*) and BRANCHED 2 (*BRC1-2*) transcription factors, which are important centres of signals controlling the ability of a bud to grow out, revealed altered shoot architecture and increased bud outgrowth (Muhr et al. 2018). Recent studies also reported successful CRISPR/Cas9 mutational efficiency for poplar flowering genes and a large mutation dataset (Elorriaga et al. 2018; Bruegmann et al. 2019). These studies demonstrated promising strategies for the production of lignocellulosic biomass using less land and helping the conservation of natural forests and reducing the environmental problem of pulp and paper processing.

Solar energy stored in plants is extensively used as fossil fuel in last century for energy production, which results in of greenhouse gases emission and consequently global warming. Interestingly, the same solar energy stored in plant biomass can be used as renewable, eco-friendly, sustainable, and better alternative to fossil fuels (Verma and Dwivedi 2014; Capstaff and Miller 2018). The energy found in lignocellulosic biomass can be effectively released by its biological conversion into biofuels (alcohols) using microorganisms and/or enzymes (Zeng et al. 2014). This idea was realized firstly on food crops such as corn, sugarcane and wheat which led to increase in price of food grains and other related products. This food/fuel competition entailed the usage of lignocellulosic biomass as alternative feedstocks for biofuel production. Plants grown on marginal agricultural lands such as switchgrass, miscanthus, sorghum, and poplar as well as straw producing grain crops (maize, soybean, rice, wheat, yucca, and barley) are the main sources of lignocellulosic biomass that can be utilized for the production of biofuels (Yoo et al. 2018; Wang et al. 2012). Unfortunately, crystalline recalcitrance nature of lignocellulose, heterogeneity degree of polymerization, rough particle size and protective covering of lignin create an extra expensive pre-treatment process to loosen lignin and allow polysaccharide accessibility for enzymatic saccharification and microbial fermentation (Welker et al. 2015). Therefore, it is necessary to generate easily degradable lignocellulose producer plants to decrease the requirement for pre-treatment methods. These unsuitable characteristics of lignocellulosic biomass for biofuel production were targeted in several genetic manipulation studies to obtain plants more amenable to bioprocessing. Genome editing techniques were effectively utilized to create lignin double mutants in biomass crops. TALEN, for instance, was successfully applied in sugarcane, a crop accounting for nearly 80% of sugar produced worldwide and the most important source of ethanol production. Jung and Altpeter (2016) successfully applied a TALEN-based approach to target the conserved region of caffeic acid O-methyltransferase (*COMT*) and to create multi-allelic mutagenesis in lignin biosynthesis and generate lines exhibiting a 29–32% reduction in lignin content compared to controls with significantly reduced S subunit content and elevated hemicellulose content. In another study, these TALEN-mediated *COMT* mutants were grown on field to test their survival and biomass production performance. The results of the study revealed 20% reduction in lignin content and S/G ratio, which resulted in 44% improved saccharification efficiency. Biomass production performance of *COMT* mutants did not differ significantly from the original cultivar under field conditions (Kannan et al. 2018). As mentioned earlier, Park et al. (2017) mutated a key gene (*4CL*) in lignin biosynthesis with CRSPR/Cas system in switchgrass, a model biomass species characterized by high ploidy level. The results of the study revealed less lignin content and significantly increased glucose and xylose release in knockout plants compared to control. Furthermore, another CRISPR/Cas9 system introduced simultaneous mutations at three gene loci, teosinte branched *1*(*tb1*)*a*, *b* and phosphoglycerate mutase (*PGM*), when stably transformed mesophyll protoplasts of switchgrass showing that the CRISPR/Cas9 can be used for multiplex GE and produce homozygous mutant plants in T0 generation. Interestingly, plants with *tb1* gene mutations had increased tiller numbers, which is a useful genetic material for breeding switchgrass cultivars with high biomass yield (Liu et al. 2018). Miscanthus (*Miscanthus* spp.) is a non-food, second-generation bioenergy crop and a perennial C4 grass that grows well even in marginal lands. The biomass of *Miscanthus* has a carbohydrate content of more than 60% (w/w) regardless of the variety, in which approximately 40% is cellulose based on dry weight (Qin et al. 2012). Glucose generated from the hydrolysis of cellulose is the main fermentable sugar for bioethanol production and thus the cellulose content of *Miscanthus* is a major agronomic characteristic in using this grass as an energy crop. The transcription factors MsSCM1 and MsMYB103, which were found to act as regulators of lignin biosynthesis leading to specific lignin qualities, represent interesting targets for lignin content manipulation and composition towards tailored biomass (Golfier et al. 2019). GE approaches are not documented yet for this species. CRISPR-derived C3′H-knockdown in rice indicated suppression on lignin composition, and on the assembly of other cell wall components in rice (Takeda et al. 2018). Such structural alterations in rice cell walls reportedly enhanced biomass digestibility and saccharification. Recently, hydroxycinnamoyl transferase in *Arabidopsis* was targeted by fibre-specific promoter based Cas9 (Liang et al. 2019). This study revealed that xylem-specific Cas9 expression is able to reduce lignification in xylem cells by avoiding defects on pleiotropic growth of full knockout *Arabidopsis* mutants.

The increasing demand for biofuel production leads to an increased demand for the raw plant material, and it has been a driving force for plant researchers to create plant feedstocks tailored for biodiesel production using either classical or modern breeding tools for the creation of oilseed varieties with higher oil content and optimal fatty acid composition for biodiesel production. Biodiesel is a fuel composed of mono-alkyl esters of long-chain fatty acids derived from biomass from plant oils, which consists mostly (> 95%) of triacylglycerols (TAGs) and short-chain alcohols. Another source of biodiesel that reduces its cost is waste vegetable oils and non-edible crude vegetable oils. Currently, non-edible oil yielding plants for the second-generation biodiesel production include *Jatropha*, castor bean, cotton, Pongamia, tobacco, mahua, neem, and *Camelina*. Plant oils derived mainly from TAGs in seed tissues (embryo or endosperm) represent a promising source of renewable biofuel. For most of these feedstocks, agronomic and crop production improvements are just beginning to be applied through GE approaches. Oilseed rape (*Brassica napus*) is an annual crop native to the Mediterranean region and Asia that produces seeds with an oil content of about 40–45%. The CRISPR/Cas9 system was used to target *FAD2* gene, which encodes an enzyme that catalyses the desaturation of oleic acid, creating mutants with statistically significant increase in the oleic acid content compared to that present in wild-type seeds (Okuzuki et al. 2018). Jatropha (*Jatropha curcas*) is another promising plant for biodiesel production due to its high oil content in seeds. Several studies suggested that exogenous cytokinin treatment can significantly increase the total number of flowers per inflorescence, the female-to-male flower ratio, and the seed yield (Fröschle et al. 2017; Pan and Xu 2011). The CRISPR/Cas9 system was used to study the function of cytokinin metabolic gene CYP735A gene and found that the concentrations of trans-zeatin (tZ) and tZ-riboside decreased significantly in the gene mutants, which showed severely retarded growth (Cai et al. 2018). In camelina (*Camelina sativa L*.), an allohexaploid species of the *Brassicaceae* family and an oilseed crop for biofuel production, Cas9 and a sgRNA targeted all three diacylglycerol O-acyltransferase 1 (*DGAT1*) or phospholipid:diacylglycerol acyltransferase 1 (*PDAT1*) homeologs simultaneously, which are important genes for triacylglycerol biosynthesis. The resulting mutant lines reduced seed oil and altered fatty acid composition. This application demonstrated the ability of the technology to target all three homeologs simultaneously in this species (Aznar-Moreno and Durrett 2017). In another application of CRISPR/Cas9 technology in the same species, three alleles of the fatty acid elongase1 gene were targeted aiming to reduce the amounts of very long-chain fatty acids (VLCFAs) and improve fatty acid composition in seeds. VLCFAs were reduced to less than 2% of the total fatty acids compared to over 22% present in the wild types (Ozseyhan et al. 2018).

Lignocellulosic biofibres have attracted a renewed attention in recent years due to their low production costs, decomposable nature, proper physical properties, and environmental friendliness. Biofibre producing plants such as herb, cotton, jute, and flax have important utilization potential in biomedical science, textile, and automotive industry. Although it improves mechanical strength of the biofibre, the presence of lignin in lignocellulosic biomass of these plants decreases elastic properties of fibres and utilization, especially in textile industry. Therefore, genetic manipulation on fibre plants to obtain low-lignin fibres with improved elastic properties is the most desirable target for bioengineers. Cotton plant is the most important biofibre producing species in that group due to the importance of its fibre and derivatives for our daily life and the world economy. Therefore, GE with CRISPR mutational efficiency on cotton has been tested and successfully established recently. These studies revealed a moderate to high gene editing efficiency for both exogenous transferred genes (Chen et al. 2017; Janga et al. 2017) and endogenous genes in cotton (Chen et al. 2017; Gao et al. 2017; Li et al. 2017b; Wang et al. 2017, 2018). Li et al. (2017b) developed a CRISPR/Cas9 system in cotton by targeting *GhMYB25* genes functional in lignocellulosic fibre formation and elongation. A similar system was also applied in cotton to mutate alanine-rich protein (*ALARP*) gene, which encodes an alanine-rich protein that is preferentially expressed in cotton fibres (Zhu et al. 2018).

## Future perspectives: Can plants lead the way in Europe and beyond?

Development of new breeding techniques, such as GE, could provide new perspectives for more efficient plant breeding. In crops, most of the agronomically important traits are complex phenotypic traits controlled by polygenes, and it is usually necessary to study more than a single gene or single class of genes to understand molecular mechanisms underlying respective traits. GE tools could be used by breeders to evaluate and validate the strength of the predictive breeding value of a given candidate gene by easily transferring its best alleles into different genetic backgrounds (Nogué et al. 2016). As different genes can be individually engineered at the same time, GE also provides the means for modification of linked genes or QTLs that are usually difficult to segregate due to the limitations of meiotic recombination (Flavell 2010).

As in genetic transformation, regeneration efficiency could be a bottleneck for the effective deployment of GE techniques in crop breeding (Miladinović et al. 2019). Many crops such as cotton and sunflower are either recalcitrant or have difficult and long transformation protocols (Taški-Ajduković et al. 2010; Gao et al. 2017). Furthermore, since regeneration capacity is genotype-dependent, in crops where a transformation method has been established, such as sorghum, many of the elite varieties remain uncooperative, not being amenable to transformation (Botella 2019). In addition to the already-mentioned problems, in polyploid crops the GE protocols should enable simultaneous targeting of multiple alleles, which could be an obstacle for GE of wheat and other crops with complex genomes. Thus, it is crucial to determine the efficiency of the sgRNAs selected for the CRISPR/Cas9 system in advance, as the sequence of the target site has a strong influence on the efficiency of the sgRNA (Wang et al. 2014). Agroinfiltration is one of the methods that has been used for CRISPR/Cas9 target validation in hard-to-transform crops such as cotton (Gao et al. 2017). In tomato, DNA constructs for ZFNs were introduced by electroporation of seeds (Hilioti et al. 2016).

Another aspect that could potentially affect application and potential impact of GE, as well as its public acceptance, is the choice of agronomic or quality traits to be either improved or introduced. In most of the crops, the choice of the traits to be improved, either by classical breeding of genetic modifications, was mostly technology-driven, taking into account the needs and benefits to farmers, processors, and distributors. This especially stands for vegetables, which were constantly selected for improved “shelf life” and shipping quality that created varieties such as “cardboard” strawberry and bouncing tomato (Georges and Ray 2017). Combined with the misconception that all these flavourless varieties are produced through transgenic approaches, this leads to discrepancies in acceptance of the new varieties by farmers and industry on the one side, and final consumers on the other side. Hence, when choosing the crops and traits to be improved by GE, one has to bear in mind that the public will accept new technology only when individuals decide for themselves that products obtained with the use of new breeding tools will contribute to their personal well-being.

These further emphasize the importance of the need for keeping the public well informed, since, based on the past experiences when experts and consumers have disagreed, the opinions expressed by experts might not override consumer perspectives in any meaningful way (Lassoued et al. 2019). Hence, it is the responsibility of scientists to keep an adequate flow of information in a manner that would promote an informed public understanding of the goals and means of GE, while providing a clear account of risks vs. benefits, as well as emphasizing the risks of opportunities lost (Miladinović 2020). Such interaction with society may prevent the spread of misinformation.

Finally, although GE editing approach is superior and much more precise than classical genetic modifications, it will likely face similar challenges depending on how governments perceive the technology. So far, many countries have indicated that if no foreign DNA is present in a crop variety, it will not require any additional regulatory oversight or risk assessment, which is in contrast to the EU judgement that even in the absence of foreign DNA any genome-edited variety must be regulated as equivalent to transgenic GMO varieties (Lassoued et al. 2019). This will ultimately contribute to shaping the public perception of GE, but also affect its application and impact in the EU.

Overall, plant GE is a powerful tool in the development of novel plant species with desired agronomic traits and nutritional value. Developing GE elite plants carrying targeted gene mutation(s) without foreign DNA may help increase public acceptance of agricultural products and free them from regulatory monitoring in order to advance their use in plant breeding programs and commercial-scale production. Plant systems are relatively economical to maintain with short generation cycle and ease to be handled compared to animals, and hence, they can be particularly attractive for testing new concepts and develop new approaches in GE-related research that aims to improve accuracy, versatility, and efficiency of these molecular tools and apply them with ease to other systems. However, the lack of regulations and uncertainty about possible applications could affect implementation of GE for plant improvement. The researchers have knowledge and resources to apply these new tools for introduction of new traits into plants, but for the time being this research is generally put on hold, waiting for new regulations and proper assessments of GE methods used for plant improvement. Gene targeting in plants plays an important role in providing new information on genetic and genomic analyses, gene networks and single gene variant function, which in case of well-conserved genes across living species may advance our understanding on the cellular and molecular workings of advanced eukaryotic species and at the same time pave the way for development and implementation of new, more efficient breeding tools, and shorter time needed to transfer the knowledge from laboratory to field.
